# Medical News and Miscellany

**Published:** 1892-11

**Authors:** 


### Medical News and Miscellany.

Dr. H. H. Beverly has changed his residence from Prilgrim Lake to Smiley, Texas.

Dr. R. T. Morris proposes to circumcise females for obscure troubles supposed to be reflex, and caused by adherent prepuce. Fact. See *Medical Mirror.*

Dr. I. N. Love, the only genuine and original, uses the personal pronoun, first, singular, in his editorials instead of the time honored "we."

Good Gracious!—"They" have started a journal in Chicago called the Journal of Orificial Surgery,—to fill a long felt want. Evidently "they" saw an "opening."

Errata.—In Dr. E. B. Jackson's article on gonorrheal affections in women his prescription for vaginal injection should have read "chloride zinc, gallic acid, aa 15 grs. to *eight* ounces of water" instead of to "one ounce" as printed.

Attention is called to the new advertisement of the Canton Surgical and Dental Chair Company in this issue. Their chair is very popular, so much so that their gentlemanly agent, who passed through Austin last week, complained that he found many of the physicians hereabout already supplied with one.

Dr. T. J. Bell, of Tyler, Texas, has been elected one of the Vice-Presidents of the Pan-American Medical Congress to meet in Washington in September, 1893, and Dr. H. Leonard, of New Braunfels, is a member of the general committee of ways and means. Dr. Carhart, of Lampasas, is Assistant Secretary-General.

The number of matriculates at the Texas Medical College is twenty-three, one more than last year, though there are several students present who have not yet taken out tickets. The JOURNAL learns that some very interesting work is being done in the Physiological Laboratory, and that Prof. Keiller has made some lovely "dissections."

News from Texas.—The sugar planters on the Brazos are "raising cane." The engineer on the new street railroad says the dummy will not answer; the dummy people are mute on the subject, but the land agents are talking lots. The drummers are taking march orders, while the merchants cancel orders for goods they can't sell, looking for a fall this fall.

---

Dr. David Cerna, late of Philadelphia, has been chosen by the University Regents to fill the auxiliary chair of Demonstrator of Physiology, and Dr. Geo. P. Hall, the well known oculist of Galveston, has been appointed Lecturer on Diesases of the Ear, Nose and Throat in the Texas Medical College at Galveston. Dr. R. C. Hodges remains as Lecturer on Diseases of the Eye.

---

At the recent meeting of the American Orthopedic Association, held in the city of New York. September 20, 21 and 22, 1892, the following officers were elected to serve for the ensuing year: President, Dr. A. J. Steele, New York; Vice-Presidents, Dr. Samuel Ketch, New York, and Dr. Arthur J. Gillette, St. Paul; Treasurer, Dr. A. R. Judson, New York; Secretary. The next annual meeting will be held in St. Louis the third week in September, 1893.

---

The Committee on Organization of the Pan-American Medical Congress will issue the preliminary announcement of the Congress within a few weeks. This anuouncement will show that the organization has been perfected in almost every colony and country of the Western Hemisphere. The local medical societies in each of the constituent countries are made auxilliary to the Congress, which will be held in Washington, D. C., September 5th, 6th, 7th, 8th, 1893.

---

Married.—The JOURNAL acknowledges the courtesy of the following:

"Mr. and Mrs. J. E. Wallis invite you to the wedding reception of their daughter, Henry Pearl, and Dr. Robert W. Knox, on Thursday, November the tenth, eighteen hundred and ninety-two, at half past five o'clock. 1502 East Avenue I, Galveston, Texas."

The JOURNAL extends hearty congratulations to the handsome young doctor.

The U. S. Pharmacopœia "1890," which will be published during 1893, adopts in great measure the *metric system* of weights and measures; this will doubtless create much confusion in the minds of physicians and druggists, and lead to many misunderstandings and errors. In order to provide a guide for the proper dosage, etc., Dr. Geo. M. Gould, author of "The New Medical Dictionary," has prepared a very complete table of the official and unofficial drugs, with doses in both the *metric* and *English* systems; this table is to be published in P. Blakiston, Son & Co.'s Physician's Visiting List, for 1893, together with a short description of the metric system.

---

The American Public Health Association—Meeting in Mexico, November 29 to December 3d. The only requirement for membership is a good moral character, an interest in hygiene, and the endorsement of two members. Blanks will be furnished at the meeting, A special Pullman train for doctors will pass through Austin on the morning of November 22d, and will lie over one day at San Antonio. Fare for round trip from Austin, $38.25; other places in same proportion. Tickets on sale at all offices. Good till December 31, with "lay over" privileges at points of interest. For further particulars address General Passenger Agent, Maj. Lewis, Driskill Hotel, Austin.

---

Migratory.—The *Southern (?) Journal of Homeopathy*, late of New Orleans, late of San Antonio, late of Austin, Texas, is now published at *Baltimore.* It seems it can't stay lit, but like Dr. Willis King's branch-water man, is always looking up "a good place to move to." Fisher started it in Austin, moved it to San Antonio, thence the publishers moved it to New Orleans; and now, to accommodate the new editor, it has been moved to Baltimore. So, the "So-Journ"(1) does not *so-journ* long in any one place. It is about time to drop the "Southern," and call it the "migratory" or "peripatetic" or "itinerant" journal of homeopathy.

---

Dr. R. E. Moss has removed from Mexia to San Antonio, and having fitted himself by two special courses on the eye, ear, etc.,—one with the celebrated Knapp,—in addition to his general knowledge, has abandoned the general practice, and will devote himself exclusively in future to diseases of the eye, ear, nose and throat. The doctor is a courteous gentleman, and has a

State wide reputation as a surgeon and physician. Moreover, he is personally very popular, and we predict for him and wish him a splendid career in his new field. His many friends should remember him when they have special cases for the oculist's work. His address is 203 Alamo Plaza, San Antonio.

---

**Careless Writing.**—Perhaps one of the most common faults with some really learned writers is a disregard of the rules of syntax. It is a common thing to encounter, in almost any of our exchanges, just such a solecism as this (by one of the ablest Philadelphia professors): "I am told by many that they have used this method which I have condemned with great success." If he had condemned the method with great success, it appears to us "they" would have ceased to use it. At a political meeting, recently, an able ex-district attorney, in relating an anecdote, said, "he cut his knee hunting 'possums with a hatchet." That is a new way of hunting' possums. The above reminds us of the man who bought a cow for his wife with a blaze face, and a pony for his little daughter with a silver mane and tail, and also the man who dug a well with a Roman nose.

---

**For Sale.**—An opportunity to have a healthy, quiet and comfortable home, with a regular practice from the start of $2000 a year in a section of Texas that has the combined resources of the grain of the North, cotton of the South, and cattle of the West.

Churches, school, Masonic lodge, cheap lands, a good opportunity to embark in the stock business. A bonanza for the wornout practitioner of the malarial districts who is seeking health, and wishes to continue in practice. Improved acre lot, dwelling four rooms, two porches, chimney, cistern, garden, orchard, out-houses, and well, desirably located. Opposition weak. Terms, $1000. Will remain till purchaser is thoroughly satisfied he can hold the field. Object, post graduate course.
Address, W. B. ANDERSON, M. D.,
Content, Runnels county, Texas.

---

**Vive la Homeopath.**—That the ignorant and credulous should be deceived by the pretensions of the homeopaths is not surprising; but that intelligent, cultivated persons, even shrewd lawyers, should have any confidence in their pretensions is indeed remarkable. Mrs. Harrison in her last and long illness, was under the care of a homeopath. (It is true, a distinguished regular of New York was in consultation,—just how he reconciles it to his conscience is not clear to understand.) General McClellan, with an attack of neuralgia of the heart, was in the hands of a homeopath, who let him die, when, no doubt, a hypodermic of morphia and atropia would have relieved him; the newspapers at the time stating that the remedies of the "doctor" had no more effect than those recommended by the house-hold members, "domestic" remedies; and Gov. Fifer, of Illinois, has appointed as surgeon-general of the State a homeopathic fledgling, on whose diploma the ink is said to be hardly dry.

---

**Exterpation of the Uterus for Cancer.**—Dr. Charles A. L. Reed, of Cincinnati, presented to the recent meeting of the American Association of Obstetricians and Gynecologists a report of twenty-five cases of complete vaginal extirpation of the womb for cancer with only two primary deaths—one from shock and one from iodoform poisoning. Of the twenty-five operated upon but fourteen were of more than two years standing, and hence were all that could be discussed with reference to their ultimate results. These fourteen were divisible into two classes of seven each, viz: those in which the disease had existed for more than six months before the operation and those in which it had existed for less than six months before the operation. Of the first class, i. e., those of more than six months (an average of 10+ months) previous duration, all were dead; of the second class, i. e., those of less than six months (an average of 4+months) previous duration only one has since died. One of the recoveries is of more than five years duration. The conclusion from these figures is that cases of cancer of the uterus ought to be remanded for operation as soon as diagnosed. Dr. Reed looks upon total extirpation as the only operation to be advised or practiced in those cases, the primary mortality from which, in experienced hands, varies from five to eight per cent.

---

**Alvarenga Prize of the College of Physicians of Philadelphia.**—The College of Physicians of Philadelphia announces that the next award of the Alvarenga Prize, being the income for one year of the bequest of the late Señor Alvarenga, and amounting to about one hundred and eighty dollars, will be made on July 14, 1893, provided that an essay deemed by the Committee of Award worthy of the prize shall have been offered.

Essays intended for competition may be upon any subject in medicine, but cannot have been published, and must be received by the Secretary of the College on or before May 1, 1893.

Each essay must be sent without signature, but must be plainly marked with a motto and be accompanied by a sealed envelope having on its outside the motto of the paper and within it the name and address of the author.

It is a condition of competition that the successful essay or a copy of it shall remain in possession of the College; other essays will be returned upon application within three months after the award.

The Alvarenga Prize for 1892 has been awarded to Dr. R. H. L. Bibb, of Saltillo, Mexico, for his essay entitled:—"Observations on the Nature of Leprosy."

CHARLES W. DULLES, Secretary.

---

**Death of Dr. Asa Pope.**—The many friends of the several doctors Pope, will be much pained to learn of the death of Dr. Asa Willie Pope. He was immensely popular, and universally beloved by all who knew him. Courageous in the discharge of every duty, a man of unflinching nerve and of unwavering integrity, he was, with all, as gentle as a woman, and at times modest to embarrassment. He died at his home in Marshall, Texas, Thursday, Nov. 3rd, inst., at 11:20 a. m., of typhoid fever, after an illness of about thirty days.

"Dr. Pope was born in Washington, Georgia, Feb. 17, 1858. His father, the late lamented Col. Alexander Pope, moved to Marshall the same year. The subject of this sketch was educated at Marshall and at Roanoke College, Salem, Va. At the latter place he graduated, and, while being in a four-years class, finished the course in two years, taking third honors. In 1882 he graduated from the Medical Department of the Tulane University, New Orleans. He then came to Marshall and engaged in the practice of his chosen profession, with a success never before attained by any physician of his age in Marshall. When he was taken with the sickness that ended his life, he stood very high as a physician and had, perhaps, as large, if not the largest, practice of any physician in Marshall. He stood equally as high as a gentleman, a citizen, and an honest man.

"Dr. Asa W. Pope was married May 19, 1891, to Miss Bessie Parmele, at the home of her parents, near Valley Creek, Fannin county, Texas. He leaves her to mourn his loss, with a son, aged three months."—(*Marshall Paper.*)

It is gratifying to his friends to know that he was conscious up to the very moment of his death, and perfectly reconciled to the inevitable. His death was peaceful and quiet, as had been his life; he suffered no pain. Always delicate, physically, in later years he had limited his practice almost entirely to office consultation. His early death is a great loss to the medical profession of Texas.

---

### A PROPOSITION TO PHYSICIANS.

Rockport is beyond question the ideal point in the South for a Sanitarium. The company owning the Aransas hotel desire to turn it over to physicians to be converted into such Sanitarium.

The hotel is comparatively new, being only three years old, has one hundred fully furnished rooms and complete outfit of office, dining room and kitchen furniture and appurtenances. The hotel could be used without a single change for a Sanitarium, Hospital or Keely Institute. The building fronts the bay, has one of the loveliest views in the world, and is located in an air and climate that for health is simply incomparable.

Catarrhs, bronchitis, nervous prostration, insomnia, liver complaints, etc., are either cured outright or greatly benefited by a month or two of residence here.

The recreation to be had in fishing and hunting is unsurpassed on the continent. Red fish, trout, tarpon, Spanish mackerel, porpoise, sheep head, etc., abound, and bite splendidly. In the winter every imaginable water fowl, such as the duck, goose, brant, swan, curlew, snipe, beautiful pink flamingo, etc., fill the bays, shores and inlets in countless thousands.

An invalid could amuse himself every hour in the day while getting well. The hotel has its own system of water works, is lit by electric light, and occupies the central and largest block in the city. With a small expenditure, hot salt water baths could be introduced into the hotel for curative purposes. Fine sulphur wells are to be had by boring.

Address for further propositions or terms,

THE ROCKPORT HOTEL CO,

Rockport, Texas.

